# "The Hospital" Nursing Mirror

**Published:** 1901-03-30

**Authors:** 


"The Hospital" Nursing Mirror.

Being the Nursing Section of "The Hospital."

[Contributions for this Section of "The Hospital" should be addressed to the Editor, "The Hospital" Nursing Mirror, 28 & 29 Southampton Street Strand, London, W.C.]

### Notes on News from the Nursing World.

### TWENTY MORE NURSES DESPATCHED TO SOUTH AFRICA.

The Secretary of State for War informs us that twenty more members of the Army Nursing Service Reserve have left England for South Africa in the s.s. *Bavaria*, namely, Misses L. Allman, K. A. Cartwright, E. L. Chadborn, J. M. Clay, A. Cook, G. Johnston, A. L. Jones, J. McCotter, A. Paget, J. Paget, M. L. Potter, E. A. Powner, B. Rennie, M. J. Robertson, A. Roper, A. G. Symonds, M. Terry, L. Thornton, E. Townsend, and M. L. Tyndall. Miss Mary Louise Potter was trained at Paddington Infirmary. She was afterwards staff nurse at the Royal Infirmary, Edinburgh, and sister at Poplar and Stepney Sick Asylum. Since July, 1897, she has been at Guy's Hospital. Miss Elizabeth Annie Powner was trained at Hereford General Infirmary. She has since been staff nurse and head nurse at the Victoria Hospital, Folkestone, and head nurse at St. Bartholomew's Hospital, Rochester. Miss Bathia Rennie was trained at the Royal Infirmary, Aberdeen, and was afterwards staff nurse in the same institution. She has since been charge nurse at the Convalescent Home attached to the Aberdeen Infirmary, and Queen's district nurse at Brechin and Uddingston, N.B.

### NURSES AND THE CENSUS.

The development of the nursing movement is demonstrated by the instructions given for filling up the columns headed "profession or occupation" in the census schedules. The term "nurse" by itself is described as too indefinite. The kind of nurse is to be stated, as "hospital nurse," "sick nurse," "monthly nurse," &c. But whilst the authorities complain that the public are too vague in the description which they give of their occupations they themselves manifest signs of the same want of definiteness. The distinction is not between a "hospital nurse" and a "sick nurse," for they are both sick nurses. The real distinction, and the one which would have been of interest, is between the "trained nurse" and the "untrained nurse." If this distinction had been made the result might have been an interesting record of the number of trained nurses pursuing their calling in the United Kingdom, and of other persons who attend the sick without any special qualifications for the task.

### CO-OPERATION AMONG OLDER NURSES.

Under this heading last April we gave an account of a new co-operative society of nurses in process of formation, in connection with the Royal British Nurses' Association. The essential feature of the undertaking was the rule that no member was admitted to the staff below the age of forty. It thus became a standing protest against the early age limit imposed in nursing as in many other occupations open to women, and cut at the root of the doctrine that after forty a woman's best working time is over. The co-operation was opened on January 1st at 10 Orchard Street, W., as the Auxiliary Nurses' Society, and it is already showing signs that it meets a public want. The provision of permanent nurses for chronic cases has long been felt as a difficulty in the ordinary nursing institutions, where the nurse is permitted to remain two, or at the most three, months at each case. Every time the nurse is changed, for a chronic invalid, the household experiences a worrying upheaval and the patient suffers severely; yet when nurses fresh from hospital are employed for long cases a frequent change is inevitable or the nurse would prove restless and her work would deteriorate. When nurses have had some experience in private work they are very glad to be settled for a time, and a year or two does not seem to them too long to devote to one patient. Very touching friendships often spring up in this way between invalids and those who have the care of them, and the forlorn sensation of being merely a "case" fades away in the security of mutual regard. If the auxiliary nurses can meet the demand for permanent nurses endowed with hearts as well as with heads, they will do good public service. There is evidence also that the older nurses will be cordially welcomed in many cases where the presence of young women is undesirable. Several are now comfortably installed at cases in which very competent nurses were found unsuitable, solely on account of their youth. It is true that not all nurses over forty are capable of a high order of work. Defects of character and training, which were not so very perceptible when youth and novelty lent enthusiasm to duty, manifest themselves unpleasantly as years go on. Herein lies the difficulty for those who have reached their prime, and can add to other high qualities this also, that they have learnt to bear the yoke. Their work is more steadfast and self-forgetting than in the years that have gone before. Their presence brings relief to the distressed household, and consolation to the dying. Yet because of their unworthy sisters they are often called in question and set on one side. To put them in their right place and make their services readily accessible to the public is the object of the Auxiliary Nurses' Society, and to this admirable work we cordially wish the utmost measure of success.

### THE NURSING VOCATION.

The opinions of the author of the article on "The Nursing Vocation" in another column are worthy of consideration, for her experience has been unusually long and varied. In a private letter she says, "Though I am no longer young, I still earn a salary of £100 a year. I have been poor, I have had a struggle. But I began with the earnest wish to work well, to be a good nurse, and I never believed in the words 'can't' or 'menial.'" Further on, she says, "I think the majority of patients friends are generally so grateful for the help a good nurse is to them that they will do anything to make her happy, and we who have to help the sick and suffering ones, be they the rich or the poor, ought to sink ourselves and our dignity in the consoling thought that we are doing our very best in our own corner. I would not have nurses neglect their rest time, walk, or proper food, but I would like many of them to be less aggressive in respect to their rights."

### A BELGIAN NURSE IN THE TRANSVAAL.

The experiences of Madame Alice Bron as a nurse in the Boer Army were so unpleasant that, as our readers know, she has changed sides, and is now employed in nursing the English. In narrating her adventures in the Transvaal last year Madame Bron again contrasts the conduct of the Boer with that of the British patients to the great disadvantage of the former. Of her first consignment of forty English sufferers from fever and wounds she says: "These forty soldiers of the enemy's conquering army under the care of a single nurse behaved themselves admirably without an equivocal word or gesture, being almost too much afraid of putting me out to ask me to give them water, and always with 'Please, Sister,' or 'Thanks, Sister,' muttered in a tone of gratitude and respect." When the Boers found that Madame Bron was occupied in nursing the English, they knocked off her supplies, with the result that she fainted from starvation on the bed of an English officer whom she was attending in the Orange State Hospital at Jacobsdaal. On this being known, however, the General sent her word that although the Orange Free State had undertaken to provide for all the hospitals, she might draw what she pleased from the English stores; the chaplain brought her a chicken, the German doctor chocolate, and her own wounded tried to divide their rations with her. During the three months she gave her services gratuitously to the wounded Boers, she only once received words of gratitude from Boer lips.

### MORE NURSES WANTED AT LEICESTER INFIRMARY.

It will be seen from an article in another column that while the nursing arrangements at Leicester Infirmary are generally satisfactory, the staff is not large enough for the needs of the patients. The lady superintendent frankly says that at least six or seven more nurses are wanted, and she makes good her case by the citation of the fact that in wards containing thirty beds there is only one night nurse on duty. Seeing that the infirmary is the only hospital in the town which depends exclusively for maintenance upon voluntary support, and as a great many cases come from the county, there ought to be no difficulty about funds. The excellent work done by the nurses seems to be appreciated, but it would lighten the burden of the lady superintendent and her staff if new subscriptions were sent in to take the place of the old ones which have fallen off. It is certain that the town will continue to develop, and the strain upon the infirmary will therefore become greater instead of diminishing. In the new wing, which is about to be built, there will apparently be no further accommodation for nurses. But is it impossible to remedy this? The very addition of a wing will mean more work for the nursing staff, which therefore ought to be augmented when the enlargement is completed.

### THE SWINDON GUARDIANS AND THEIR HEAD NURSE.

Nurses, of course, are not immaculate, and a not incredible accusation is made against the head nurse at the Swindon Workhouse Infirmary. In the report of the house committee which was considered at a meeting of the Guardians the other day, it was stated that complaints were made by the other nurses that "very much unpleasantness and discomfort were caused by the head nurse in her overbearing and exacting demands, and her practice of rebuking them in a fit of passion even before the inmates of the ward." This, they said, "often occurs," and in support of their allegation the master reported that one of the nurses had left a week before the expiration of her notice, "owing to friction with the head nurse." The Guardians having discussed the question at some length, it was decided to ask the medical officer to inform them if he had any specific charge to make, because the complaints of the staff were of too general a character to apply for the intervention of the Local Government Board. We offer no opinion on the merits of the accusation, to which there may be a complete answer, but the incident affords another proof of the unsatisfactory working of a system which admits of divided authority.

### FRICTION AT BRADFORD.

A very unusual complaint has been made by the Rev. C. W. N. Hyne, of Bradford. At the annual meeting of the Bradford Incorporated Nurses' Institution, which was otherwise of a satisfactory character, Mr. Hyne, in proposing a vote of thanks to the retiring committee of the District Nursing Branch, said that "the medical men in the particular part of the city in which he lived did not recommend the district nurses. In cases where the friends of a patient thought a nurse was desirable they had to use a certain amount of pressure in approaching doctors on the question." The Mayoress of Bradford, who is president of the District Nursing Association, asked Mr. Hyne "if any medical man had any complaint to make against the nurses," and he replied, "I make no complaint whatever about the nurses. I am complaining of medical men." The Mayoress then confessed her anxiety to ascertain "why the medical men are acting in this curious manner," and Dr. Goyder said that he would be reluctant to believe that such a condition of affairs prevailed in any other part of the city. We agree with the Mayoress that the matter requires to be fully investigated. Not only in Bradford, but all over the country, doctors and nurses work, as a rule, in harmony, and when this is not so individuals are invariably to blame. It is obvious that where there is friction of the kind suggested by Mr. Hyne, whoever may be in fault, the patients suffer; and a remedy should be speedily found.

### HOW TO ASCERTAIN WHETHER A NURSE IS TRAINED.

Not for the first time, the risks which the public run when they employ nurses without any knowledge of their training, are pointed out by "A Trained Nurse," who in our correspondence column gives a picture of the manner in which a large nurses' home is conducted. She does not allege that there are no fully-trained nurses attached to it, but she says that "all the nurses, whether certificated or otherwise, take out printed rules with the fees charged for different diseases, and all demand the same fee and get it as a rule." "Is this," she continues, "fair, either to certificated nurses or to the public?" Of course, it is not, but there is a simple remedy, at least for the public. When a nurse is sent into a private house from a home, she should be courteously requested to mention her name and training school, and if a reference to "Burdett's Official Nursing Directory," proves that her statements are accurate, well and good. If not, further enquiry should be made. The Directory is not only a protection to the public, but it is also an obvious assistance to the trained nurse whose name appears in its pages.

### RETROGRESSION AT WORTHING.

At the annual meeting of the Worthing and District Nursing Association it was announced that the committee had decided to give up the work in the parish of West Tarring. They have arrived at this decision because, owing to the growth of Worthing and the two adjoining parishes, it is no longer possible for two nurses to cover the entire district, and also because no subscriptions have of late years been forthcoming from West Tarring. If it were not that there is a large working population in West Tarring we should only say that the parish was rightly served. Unfortunately, those who cannot afford to subscribe will suffer for the illiberality of those who can. Perhaps when the latter, of whom there are not a few, realise that unless they choose to contribute their fair share towards the support of a nurse they must go without one, they will recognise their obligation to their poorer fellow-creatures. It was hinted at the meeting that if "there was any endeavour on the part of West Tarring to help the Association in the way even of small subscriptions" something might be done; and in the circumstances we cannot blame the Worthing Nursing Association for the course they have regretfully determined to adopt.

### THE PROTEST OF A SUPERINTENDENT NURSE.

The new superintendent of nurses at Plymouth Workhouse Infirmary has made a spirited protest against the allegation that patients in that institution receive insufficient attention; and of certain questions which have been asked, such as, "Do the nurses really sit up at night?" or, "Do the nurses sleep?" she refuses to take any notice at all on the ground that there cannot for one moment be any doubt upon the matter. "A nurse's life," she adds, "at the best of times in a workhouse infirmary is indeed a trying one; and when the nurses are earnestly endeavouring to discharge their duties to the utmost of their ability, surely it is our duty in return to support and protect them." With these sentiments we cordially concur; but the principal contention respecting the nursing at Plymouth Workhouse Infirmary in the past has been that the nursing staff was inadequate, not that individual nurses were either wanting in devotion to their duties, or incapacity to perform them. With two additions, and with a superintendent who is as much alive to her responsibilities, as Miss Holliday seems to be, there ought, in the future, to be no complaints that the patients at the infirmary are not sufficiently well cared for.

### STEPNEY WORKHOUSE.

The Stepney Guardians met last week and appointed a committee to enquire, with closed doors, into the case of alleged neglect of a woman in one of the infirm wards in the workhouse. This is a mistake. The question is one of public interest, and the investigation of the Guardians should not have been private. We are glad to hear that the doctor is not only anxious that the whole thing should be thoroughly sifted, but also that a night sister should be appointed. How in the face of the statement of Miss Owen last week, that acute cases come into the wards when they cannot be taken into the sick asylum "across the way," there can be the slightest hesitation in the matter we cannot understand.

### A COOK APPOINTED AS NURSE.

We shall not be surprised if the unpleasant notoriety achieved by the authorities of Stepney Workhouse is rivalled by those of a workhouse in Norfolk. The Deerham Guardians, having been informed that the workhouse is fast becoming an infirmary, and that there are cases which require constant attention, have admitted the validity of the plea that one nurse cannot perform both night and day duties. But, instead of appointing a trained nurse for night duty, they have agreed to allow a person who has been acting as cook to take the position of assistant nurse—in other words, to nurse the cases that require constant attention during the night. She is not to be blamed for wanting to try her hand at nursing, but the action of the Guardians in accepting her services will involve them in well-deserved admonition from the Local Government Board if the ex-cook, one disastrous night, fails to understand the nature of her obligations in the new character of nurse.

### HOW TO PUT AN END TO TWO EVILS.

The nurses of St. Mary's Hospital, Paddington, are directly interested in the appeal of Mr. Alfred de Rothschild, the treasurer, who has pointed out the importance of raising £25,000 in order to fulfil the condition attached to the gift of a similar sum for the completion of the Clarence Memorial wing. Mr. de Rothschild points out that the same cause which has rendered the refusal of the admission of suitable patients a daily occurrence for years, has led to all the night nurses, and some of the day nurses having to be lodged in houses in the neighbourhood; and the completion of the new wing, he adds, will put an end to "both these evils." It is, of course, an immense drawback in a great hospital like St. Mary's to have to lodge a portion of the nursing staff outside. But the arrangement now in force is the best that can be done under the circumstances.

### LUTTERWORTH COTTAGE HOSPITAL.

The people of Lutterworth did not want the Fielding Palmer Cottage Hospital, which was kindly given to them by Mrs. Palmer, but they are now realising its worth. Last year the nurse attached to it paid nearly a thousand visits, and there will soon be four patients in the hospital. Mrs. Palmer has just sent, besides other gifts, instruments to the value of £50, which are much appreciated.

### SHORT ITEMS.

H.M. Queen Alexandra has graciously consented to continue her patronage of the Nursing Sisters' Institution, Devonshire Square, Bishopsgate.—Nursing Sisters E. C. Croft and A. R. Myring are on duty on the s.s. *Pinemore*, which left Cape Town for England on the 15th of March.—Nurses who may have to appeal to the London Spectacle Mission Society, will be interested to know that the number of applicants for help from this useful charity in 1900 was 1,156. This was an increase of 132 over the previous year. There is no falling off in the financial support given to the society.—In the recent examination of probationers held at the Cancer Hospital, Fulham Road, London, at the conclusion of the lectures given by Dr. E. J. English and Mr. W. E. Miles, the first three places were obtained by the following: A. Wood, V. B Sheppard, J. Symons, the two latter being bracketed.

### Lectures to Nurses on Medicine and Medical Nursing.

By NORMAN DALTON, M.D. London, F.R.C.P., Physician to King's College Hospital.

### LECTURE VII. DROPSY (*continued*).—THROMBOSIS MYXŒDEMA.

IN the last lecture, general dropsy, which occurs in the course of heart disease or kidney disease, &c., was considered; but dropsy may affect one part of the body only. In such cases it is usually due to the obstruction of the chief vein which carries the blood away from the part, so that the blood stagnates in the capillaries, and eventually the serum passes out into the tissues of the part. The most common instance of this is the dropsy of the leg and thigh, which follows "thrombosis of the femoral vein." The word "thrombosis" means that the blood inside the vein has clotted and has filled up the whole channel of the vein so that the blood can no longer flow through it. It occurs, perhaps, most often in the femoral vein, but it may affect the portal vein, the venous sinuses inside the skull, or in fact any vein in the body. The diseases in which this clotting of the blood is likely to occur are septicœmia (particularly after delivery), typhoid fever, and anæmia; but it may take place in the course of other affections, and it is almost certain to occur if the wall of a vein be injured in any way, or be pressed upon or invaded by tumours. In puerperal cases, and in typhoid fever, thrombosis of the femoral vein is not infrequent, and nurses should always be on the look-out for it. The first thing they may notice is the presence of dropsy in *one* foot or leg. This always suggests thrombosis of the femoral vein, because in cardiac and renal dropsy both feet become dropsical. But, not unfrequently, the first symptom is pain in the groin, which should always be carefully noted. The pain is made worse by movement; in fact sometimes it is only felt when the thigh is moved, and the patient may say that he feels as if a tight string were stretched across the groin. This description on the part of the patient is a good one, because a healthy vein is elastic and stretches and relaxes according as the thigh is extended or relaxed, but as soon as the vein becomes thrombosed it loses its elasticity, and any attempt to stretch it by moving the thigh causes pain. As soon as a nurse suspects that there is thrombosis of the femoral vein she must tell the patient to keep absolutely quiet, both to avoid pain and to avoid danger; because it sometimes happens that a piece of the clot in the vein becomes loose and is carried by the iliac veins, &c., to the heart, where it obstructs the pulmonary artery and thus prevents any blood from getting to the lungs. This is an occasional cause of sudden death after delivery. When the limb becomes dropsical it must be covered up to the groin with cotton wool in order to keep it warm, and it should be carefully guarded against injury. Also, it should be slightly raised on pillows so that the foot is higher than the body. The same result may be obtained by raising the foot of the bed.

If thrombosis occur in the course of septicæmia, microbes are present in the clot in the veins and the clot quickly softens to a material like pus. This softened stuff frequently becomes loose and is carried all over the body, and wherever a piece of it lodges an abscess will form. As the lung is the first organ reached by anything travelling in the veins, abscesses of the lungs are common in these cases.

Apart from clotting of the blood inside a vein, dropsy will occur in any part the vein of which is compressed by an aneurism or tumour. For instance dropsy of the face and arms is often caused by an aneurism which springs from the arch of the aorta and compresses the superior vena cava. If the portal vein or all its branches inside the liver be blocked, ascites occurs and is then strictly a local dropsy.

Local dropsy may, also, be due to the obstruction of the main lymphatic vessels which carry the lymph away from a part of the body. This may follow parturition from the blocking of the iliac and femoral lymphatics; and it then constitutes the common "white leg." But most often local dropsy due to lymphatic obstruction is met with in the tropics, and is caused by the presence of a worm, called the *filaria sanguinis hominis*, in the lymphatic vessels. At first local dropsy due to obstruction of the lymphatics resembles local dropsy due to obstruction of the veins, that is, if the leg be taken as an illustration, it is swollen, it pits on pressure, it is pale, cool, and not very sensitive. But it is not quite so soft as in venous obstruction, and more force must be used to make it pit. Later on, however, in lymphatic obstruction, the skin becomes thickened and tough. This condition of dropsy of the legs with thickened skin constitutes true elephantiasis.

There is a disease called myxœdema in which the condition of the skin resembles that met with in dropsy; that is the skin of the face (the eyelids, nose, &c.) and of the limbs is pale and swollen. It may be distinguished from dropsy by the facts that no pitting or pressure can be obtained (except occasionally in the legs), and that there is usually a pink patch on the cheeks, like that seen on the cheek of a badly-painted wax doll. The disease is due to atrophy of the thyroid gland in the neck. The patient becomes very slow in his movements and in his speech, and eventually the intellect fails. Fortunately the disease can be cured by taking an extract of the thyroid gland. This must be continued for the rest of the patient's life, for the bad symptoms recur if the use of the extract be discontinued.

There is another condition of the skin, which is called "inflammatory œdema," in which the skin pits on pressure. It is most often met with in erysipelas, which is an inflammation of the skin. In erysipelas of the face the eyelids and the scalp pit on pressure, and in the legs and even the arms the same sign may be detected. But when œdema is inflammatory the skin is red, and not white, as in dropsy.

As a dropsical limb is colder than normal, it must be kept warm by thick woollen stockings or gloves reaching to the groin or shoulder, or by a hot-water bottle in the bed. Further, as it is not as sensitive as normal, extra care must be taken to guard it against injury. For instance, a hot-water bottle, if placed too near, may blister the skin without the patient being aware of it; or some hard object may accidentally get into the bed and bruise the skin. Nurses should also know that dropsical tissues are very prone to be infected by disease germs. They cannot, of course, penetrate the unbroken skin, but the slightest scratch or blister is most easily infected, and once the germs have obtained admission they grow with great vigour in the limb, and set up an inflammation which is frequently fatal. If, then, a nurse should detect a scratch, she should immediately cover it with any antiseptic which she may have at hand, such as boracic lint (kept wet), or boracic ointment, or iodoform ointment, &c., until the doctor has seen it. Strong antiseptics, such as 1 in 20 carbolic, should not be used, as they may injure the skin.

### To Nurses.

**WE invite contributions from any of our readers, and shall be glad to pay for "Notes on News from the Nursing World," or for articles describing nursing experiences, or dealing with any nursing question from an original point of view. The minimum payment for contributions is 5s., but we welcome interesting contributions of a column, or a page, in length. It may be added that notices of entertainments, presentations, and deaths are not paid for, but, of course, we are always glad to receive them. All rejected manuscripts are returned in due course, and all payments for manuscripts used are made as early as possible after the beginning of each quarter.**

### The Nurses of Leicester Infirmary.

### A CHAT WITH THE LADY SUPERINTENDENT.

By Our Commissioner.

Travelling between Oakham and Leicester the other morning, I incidentally asked a fellow passenger whether he knew anything about the nursing at Leicester Infirmary. The old gentleman, with a twinkle in his eye, promptly answered, "I can only tell you that if I wanted a wife there is nowhere I would sooner go to look for one than at Leicester Infirmary." It subsequently transpired that he was a visitor at the institution, and therefore was not talking from hearsay. He went on to testify to the respect with which the nurses are regarded in the great and growing manufacturing centre. The exterior of a building which has the gaol almost for its next-door neighbour is hardly worthy of such a prosperous community, but, as I afterwards gathered from Miss Rogers, the lady superintendent, it is not an easy matter to raise the funds that are absolutely necessary for the maintenance of the only hospital in the town depending upon voluntary support, and there is no money to spare for outward embellishment.

### Not Enough Nurses.

In fact, the first subject of discussion raised the financial question, for when I asked the number of beds and the strength of the nursing staff the Lady Superintendent replied, "There are 242 beds. Of the 23 wards, including the isolation rooms, three are very large, and contain 30 beds each; the others from six to 12. The number of nurses is 56."

"How many night nurses does that allow for each of the large wards?"

"Only one. We are really short of nurses, and want at least six or seven more for the patients."

"Why do you not have them?"

"Because we have not sufficient accommodation. A wing for nurses was built twelve years ago, but it is not large enough for the present staff. A new wing is about to be commenced, and it will include doctors' sitting and dining rooms, as well as new surgeries and dispensaries, with a theatre and its annexes on the top storey. But there will be no further accommodation for the nurses in the new wing."

"Will not its erection, however, give more room in the old building for the nurses?"

"A little, perhaps. But the addition will make more work, and we shall want more room for the nurses."

### The Training.

"Your course of training is for three years?"

"Yes. I think that, as a rule, the four years' system is a very good thing. Both the nurses and the hospitals benefit by it. But here there is a practical difficulty in the way, because there are few posts to give to the nurses after their third year which would be a stepping-stone."

"I see from your rules that you have 'lady probationers' and 'lady pupils' as well as probationers, and that in respect to the lady probationer, only the daughters of medical men, solicitors, clergy, and officers of the Army and Navy are eligible for election to their appointments. But are not the ordinary probationers often the daughters of professional men?"

"Many of them are. The difference is that the lady probationers pay a premium of £15, or have that amount deducted from their salaries. The same regulations apply to them as to the probationers with regard to uniform, washing, medical attendance, certificates, height, age, and work."

### The Question of Class Distinction.

"And the lady pupils?"

"While the lady probationers are required to stay for three years, the lady pupils may enter for six or twelve months. They pay a fee of ten guineas a quarter, and though the fee is not returnable they receive a salary as soon as they are put on the staff, namely, £18 for the second year and £20 for the third. They often, at the end of a year join the ordinary staff. In the wards there is no sort of distinction between probationers, lady probationers, and lady pupils, but the two latter share a sitting-room. All class distinctions are calculated to cause friction; but there is very little here. Practically, our lady probationers are on the same footing as the Nightingale probationers."

"Do you give any certificate for one year's training?"

"Yes, to the lady pupils and the pupil nurses whom we train for private nursing institutes at Birmingham and Leeds. But we have two grades of certificates, and the higher grade can only be won after the full training of three years."

"Have you plenty of candidates for probationerships?"

"More than we require. The tendency is for girls of education to apply for all the posts. Indeed, we do not take them on the staff unless they are well educated."

"Do many fail to pass the month's trial?"

"I think that the majority may be said to succeed."

### Lectures the Third Year.

"I suppose there are two medical examinations?"

"Yes; one at the end of the first year and another, more advanced, at the end of the second. Few fail to pass. Two courses of lectures are given by the medical staff and one by the lady superintendent during the first and second years of training. We have had lectures in the third year for the last two or three years, but they are not organised. I think they are of very great value to the nurses, and that they are much appreciated."

"How do you classify the staff?"

"At the end of not less than a year a probationer is called an assistant nurse, and at the end of not less than eighteen months a head nurse. The first year is really the testing time. We keep a certain number of nurses on at the end of the third year—substantially those who wish to stay. Others go into private nursing."

### Off Duty in the Morning.

"What time do the day nurses go on duty?"

"At eight, and they are off at 9.30. They have two hours off duty one day and one hour and a half the next. On Sunday they are off duty in the morning and evening alternately, and sometimes in the afternoon as well. The night nurses go on duty at 9.20, and remain till 8.45. They dine at 9.15, and have two hours and a half off in the morning. The recognised holiday of the nurses is a fortnight, but when the work permits they have three weeks. The sisters have a month."

### Separate Bedrooms.

"Is the sleeping provision adequate?"

"It is sufficient for the existing staff. Every nurse has a separate bedroom, as you can judge for yourself. The grounds are excellent for purposes of recreation. Cycling is particularly popular, and there is a flourishing club."

"Are entertainments given in the infirmary?"

"Only at Christmas. If possible the nurses are allowed to go to concerts when they wish, but the work is frequently a difficulty."

"I notice that none of the staff are off duty in the evening."

"That is purposely arranged, and I think that in a town like this where there is little to do in the evening, except in summer, the arrangement is an advantage to the nurses."

"You have no private nursing institute?"

"No; as we have not enough for ourselves, we do not supply private nurses."

THE CHILDREN'S HOSPITAL.

"There are eleven or twelve nurses attached to the Children's Hospital," continued the Lady Superintendent. "They are admitted at the age of 21, and we keep them there until they are 23. Then they come into the other wards. In past times we have taken probationers for three years' training in the children's wards and one in the adult wards. But we have abandoned the practice, because we find that it is better to give more time in the adult wards and less in the children's"

"How many beds are there in the Children's Hospital?"

"There are 42 patients, and there is a theatre."

UNIFORM COMPULSORY.

"What is your rule as to uniform?"

"It is compulsory out of doors as well as in, the only exception being when the nurses are cycling. Then they use their club dress. I have never met with any objections to the wearing of the uniform in Leicester, and I prefer it always to be worn."

"You have been lady superintendent for some time?"

"For eighteen years. I was trained here, going to South Africa afterwards, and subsequently I was at Guy's for a time. My predecessor—Miss Burt—was at Guy's."

THE GREAT WANT.

As we went over the infirmary, Miss Rogers showed me the memorial window in the chapel—itself a memorial of Dr. Buck—to Miss Burt, who reformed the place. There is another window to the memory of the late chaplain, who occupied that position for sixteen years. In further recognition of Miss Burt's work, St. John's ward was refurnished, while the Children's Hospital, with its two very pretty wards, was erected about fifteen years ago, largely through the efforts of Sir Thomas Wright, then mayor of the town. The only defect of the nurses' quarters is that several of the large and lofty bedrooms are without fireplaces. The dining- and sitting-rooms are very cheerful and comfortable. Finally, as the Lady Superintendent said when we had finished our rounds, "things go as smoothly as one can expect in an institution of this kind, and, as I hinted before, our great want is more money."

### The Nurses' Bookshelf.

THE "A1" COOKERY BOOK. By H. N. L. (London: Simkin, Marshall, Kent and Co.)

THE "A1" Cookery Book lays claim to three special merits, viz., quality, simplicity, and variety, and its claims are amply justified in the result. Within some 150 pages may be found recipes for the cooking of soups, fish, meat, poultry, game, vegetables, eggs, sauces, sweets, cakes and bread, all of these recipes being reliable, and written with a view to suiting the capacity of the least experienced of cooks. A few features of the book are:—

1. Sixty-four excellent recipes for cake and bread.
2. List of sweets and puddings, divided into three sections:
   (a) Those containing both butter and eggs.
   (b) Those containing eggs and no butter.
   (c) Those containing neither eggs nor butter.
3. The simple secret of teaching young maids how to clean silver.

The classified arrangement of sweets should prove particularly useful both from the point of view of economy and when the question of diet has to be considered. The opening pages of the book are devoted to a few practical hints on housekeeping. These strike the reader as admirably helpful to the novice, especially the list which is furnished of the proportional amount which should be expended each week on the different tradesmen's "books." The few words on silver cleaning are much to the point; we could only wish that H. N. L. could have included in the recipe a hint as to how to persuade a youthful maid to carry out its simple directions, thereby saving herself in expenditure of time and unnecessary labour. The greater part of the book deals with the dainty preparation of plain English food; but here and there, as in the many and various ways given of cooking eggs, and in the special chapter devoted to vegetables, one recognises an influence from across the Channel. Not the least commendable feature of the book is the careful index, an essential adjunct too often neglected in both French and English books on cookery. We congratulate H. N. L. on a most excellent little work, which has only to be known to be popular.

BABY MINE. By E. PAGET THURSTAN, M.D. 1900. Pp. 65 (Publishers: Ames, Heller, and Co., 184 Barrack Street, Perth, Western Australia.)

A MOTHER'S responsibilities as regards the rearing of her offspring are by no means overstated in this little brochure by Dr. Thurstan. It is no exaggeration to say that the constitution of the individual is determined during the first two years of life. The proper expansion of the lungs, the muscular development, the shape of the chest, and the habits of the tissues generally are among the chief factors which determine whether the individual is to be healthy or subsequently to become the subject of a strumous, gouty, or rheumatic diathesis. These important factors in the development of the growing child are very largely controllable by the intelligent observance of the laws of hygiene and dietetics, and the best means of ensuring their observance is by the education of the mother The author evidently realised the importance of these generalisations in undertaking this little work for the instruction of mothers. The correct feeding of infants is beset with so many difficulties that no mother can be expected to grasp the full significance and meaning of a physiological diet. This matter must be left to the doctor, who must judge each case on its own merits, and order the appropriate diet. The duty of the mother is to recognise the early symptoms of failure of nutrition or digestive troubles, and having recognised them to apply for proper advice. The neglect of early symptoms greatly aggravates the subsequent difficulties of putting matters straight. The general management of the infant is dealt with in one chapter, and the remaining chapters are mainly concerned with the question of feeding. We think that the author might, with advantage, have insisted more strongly on the advisability of accuracy in weights and measures in the preparation of the food: the slip-shod methods of the nursery require constant supervision. On page 26 the figures given are not accurate, since it is assumed that all the cream can be separated from the milk by the process of skimming. Unless the milk is allowed to stand a dangerously long time only a proportion of the cream can be removed by this method. But mothers will be well advised to study this little book, and, as far as possible, follow the advice therein contained.

### Resignations.

BOROUGH OF REIGATE ISOLATION HOSPITAL.—We understand that, owing to indifferent health, Miss L. Amcoats has resigned her position as matron of the Borough of Reigate Isolation Hospital, and that the committee have accepted her resignation with regret.

### Lectures to Head Sisters.

By a Matron.

LECTURE I.—A HEAD SISTER'S DUTIES TOWARDS HER PROBATIONERS.

"What qualities do you consider necessary to make a good head sister of a ward?"

With a view to calling out their ideas on the subject, this question was set to a class of probationers during a course of nursing lectures, and some of the answers given may be of interest, in showing you what is expected from a nurse in your position.

One puts rather quaintly: "A head sister should give her probationers a proper understanding of the work expected from them, and make due allowance for the mistakes they naturally make."

Another says: "She should be a good worker herself, if she expects her probationers to be so."

A third gives it as her opinion that the head sister should show "kindness without favouritism," and a fourth says very forcibly indeed, with perhaps a spice of aggrievedness about her answer, that "the head sister should have a special day for every extra piece of work, and see it is done, but she must manage so that it can be done, without blaming the probationer for not doing it when she has called her off unnecessarily all the time!"

One and all were unanimous in their opinion that a head sister should be kind to her probationers; but only one had the good sense to temper her statement with these words: "She must not be afraid to use her authority when necessary." Whilst another adds: "Towards the residents and visiting staff she should maintain a friendly and respectful attitude."

Without further quotations, these are sufficient to show that probationers naturally expect their head sisters to be good teachers, good workers, good disciplinarians, kind, just and methodical, and it remains for each nurse already filling that post, or hoping soon to do so, to disappoint the expectations of these eager and often enthusiastic newcomers, or to strive, day by day, to live up to their ideals. Without constant striving this cannot be done, and living up to the very best that is in us means continuous, daily effort. Before a ward sister can adequately fulfil her duties towards her probationers, she must see to it that she herself is what she requires them to be—that she should exact from herself as well as from those working under her the right amount of steady solid work needed for the comfort of her patients and the advancement of her hospital; that she should be free from petty prejudices and jealousies; that she should hold herself aloof from gossip and scandal. Character is of the first importance; without it mere cleverness will not go far; and all our instructions and exhortations will be but as the voice of one crying in the wilderness, if we ourselves are only like sign-posts—continually pointing out the path but never treading it. A head sister will find her own faults and failings as well as her virtues curiously repeated and often intensified in her probationers. They will be very largely what she is. Unthinkingly, almost, they will adopt her manner of speaking, her ways, good or bad, of doing things, her ideas of ward management, her conduct generally. Her influence is very great and will confront her when she least expects it. If she is in the habit of running down her hospital, its management, methods and traditions, she will find they in turn will be disloyal to her. If she is sulky or touchy at a word of criticism or reproof, they will resent any fault-finding from her in the same way. If she is careless about the religious duties required from her and thinks it "does not matter" whether she has prayers in the ward or not, a tone of frivolity and indifference will quickly creep in and lower the moral tone of all under her care. She must lead where she wants them to follow. The more she magnifies her office the better she will fill it. The higher she rates her responsibilities the more she will rise to what is expected of her.

All head sisters who have filled their posts for any length of time have long since grown out of the notion that responsibility is an unmixed delight. It is no such thing. It means much thought, endless worries, a great deal of uncongenial work. It also very often means becoming the scapegoat of their ward when anything goes wrong, for the head sister is held responsible for all that happens in it. Some sisters feel their responsibilities so acutely that they always look worried. That is a mistake, because worry is infectious, and fatal to successful work; but it is better to feel your position a little too keenly than to accept such a responsible post in a light careless spirit of self-confidence.

Ward sisters need to have a wider grasp of things than their probationers—a fuller knowledge of the subjects they are expected to teach than is needed in the actual performance of routine duties. They should be able not only to do things in the best and quickest way, but also to give a reason for doing so. They need to have cultivated the habit of close and accurate observation, and to have studied sufficiently to follow intelligently the doctors' treatment of their cases. If they needed patience while they were learners, they need it doubly, trebly, now they have to teach. Everyone knows how much easier it is to do a thing oneself than to show someone else how to do it. That takes time and patience, but it is the chief duty of a head sister.

Probationers come to hospitals presumably to learn; they expect it, and they have a right to be taught. Remember that the knowledge how to do their work, easy as it may seem to you, does not come all at once. You must never scold them for a technical mistake when you have not explained it to them.

When a new probationer comes into your ward, try to put yourself in her place. Recall how miserable you probably felt, the first morning after your arrival, and how you dreaded the day before you with an unspeakable dread. Very likely she feels the same, and though it is not desirable that too much sympathy be lavished upon her, still she should be treated with kindness and consideration, without too much being expected from her at first.

Do not leave her standing about dejectedly in the ward or ward kitchen, not knowing where to go, or what to do, while you hear the night report, or waste the first ten minutes of a busy day in some little bit of gossip with a friend before she goes to her ward. That sort of thing gives a new comer a bad impression at once of you and your methods of management, besides impressing her with an awkward sense of embarrassment, and of being left out in the cold. Neglect is hard to bear at any time, still harder when one is new to one's surroundings.

On the other hand, do not overwhelm a new-comer with attentions and instructions, which will only bewilder, instead of enlightening her. Give her a kindly welcome, and at once give her something quite easy to do, such as sweeping, dusting, polishing, washing up, until she has time to get used to the ward. Never try to teach a new probationer too much at first. Sisters have been known—generally those new to taking charge—to waste quite a lot of time the first day of a new-comer's arrival in teaching her how to take temperatures, pulse and respiration—how to pad splints, roll plaster bandages and all kinds of things, which, to accomplish properly, imply a certain amount of previous knowledge. It is only waste of time to do this. Neither should you allow quite a new probationer to give bedpans, until you have had time to show her yourself exactly how to do it. Without the requisite knowledge, a bad bedsore may be occasioned by doing this carelessly: some stool that should be saved will perhaps get thrown away by mistake, or the new comer contract typhoid, through ignorance of the rules for disinfection.

### The Nursing Vocation.

By a Nightingale Nurse.

I have read with much interest, some amusement, and a big share of sadness, the letters in "Everybody's Opinion" from week to week, referring to uniform, social standing, and other matters concerning the comfort and position of nurses. May I add a little to what has already been written on these subjects from personal experience ?

### Nearly Thirty Years' Experience.

My nursing days, I am sorry to say, are nearly over, and during nearly thirty years I have nursed in all sorts of positions, from Royalty to the slums. I have been probationer, nurse, sister, matron, &c., in public work, and amongst rich and poor in private life. I think, perhaps, I know something about the life. I am not a fine lady, but just the daughter of a professional man, and being the eldest girl with no mother did not get the educational advantages of my sisters. I meant to be a nurse, and became one, and when free from home duties got the best training I could, and have, with God's help, tried to do my best.

### The Question of Uniform.

In all these years I have never worn an outdoor uniform, though, in district work and in slums, and also in some ocalities, I think it is most necessary; for, in a case of sudden illness, a nurse would be known and could be called in if required. But seeing some nurses in the streets with untidy cloaks flying in the wind showing their apron, long veils with rough fringes and often untidy hair, loud talking of "shop" in omnibuses, &c., makes me very sorry that these young women belong to such a noble calling as nursing the sick. It would be far better that they had not worn the garb rather than they should forget the nobleness of their calling. It must also be very painful to many people who, having lost their dear ones, to suddenly come across the same uniform as that worn by the nurse who helped them in their trouble. I certainly do not like the practice of wearing aprons in the streets; very few nurses get a salary large enough to cover the washing of two sets, and the aprons worn in a crowded theatre or omnibus cannot be quite sanitary at a patient's bedside.

### Black-handled Knives.

When first I was a nurse I also had a little trouble over what seemed a big thing then, but such a small matter now. At my first case—a sick child—I had black-handled knives and forks and very spare meals served on a tray without a cloth. I was very sorry for myself. I said nothing about it to the parents of the child, though I complained bitterly to a dear friend who had known me in my own home. She said to me quietly, "Get interested in your patient, do your work faithfully and well, remembering that nothing can alter the fact that you are your father's daughter and a member of a noble profession. Don't dishonour your work with petty grumbles, but show in your life that you are too good for black-handled knives." I was some time with that child—a very trying case—and after all these years, the family are amongst my dearest friends, and we often laugh about the black knives. I have always had the greatest respect shown to me since, though in some cases I have dined with the family, in my own room, upon the back stairs, in the nursery, the bath-room, and once in a kitchen with a dirty-faced slavey peeping in at intervals to ask, "'As you all you wants Missus?" I am afraid I liked that the best, it was so funny.

### We Old-Fashioned Nurses.

I have never found these things hurt my dignity. I have always respected myself, made my work the first object, and generally found that the patient's friends did their best for me. I got too keenly interested in my patient to care about much beside. I often worked with no time off or for sleep, getting my reward in the feeling that I was doing all I could possibly do to ease my patient's troubles. This sounds all self-praise and vainglory; but I often think how different everything was in the old days. We old-fashioned nurses used to ask what hours on duty we had for work, what chance of learning, our two hours taken up with lectures, and many of us readily took extra duty. Now, would-be probationers ask, "What time do we get off duty, and what sort of uniform is it, &c.?" The home sister of a large hospital said to me one day not very long ago, "The old stamp of nurses, who used to come into the home to be trained, never minding what menial work they did, doing all the little details with the greatest care, self-denying, self-sacrificing, with only the one object in view, to become the best nurse possible, is fast dying out."

### The Meal Question.

It is sad that it is so; nursing is such a Christ-like employment. With many years' experience I do not think it wise that a nurse nursing a member of a family in a large house should have her meals with the servants. The nurse is often very tired, and a quiet meal with a book or newspaper is a welcome rest. She lives her life very much in the shadows of sickness or death. She hears and sees a great deal that is strictly of a private nature in a sick room, and being questioned by servants or hearing things discussed (which I know are talked about in servants' quarters), would, to say the least, be awkward for the nurse. In a hotel, where people cannot help themselves, it is quite a different matter. The nurse may not like it, but after all we can do anything that is most disagreeable when it is only for a time. The nicest arrangement when possible, is a tray in the nurse's room or patient's ante-room; and if servants are not available, then the nurse should try to fall in with the views of the patient's friends, duly considering her own health.

Certainly, upon entering the house, it is not the time to ask "What is to be done?" Wait and see. Very often, after the first fuss of a sudden illness is over, the nurse gets her meals and comforts considered, because she is looked upon as one who is truly doing her best to help them.

### Mountains and Molehills.

When I look back upon my years it is with many regrets, and if I could live it over again, how much better, wiser, more self-denying and patient I should be! How the seeming mountains shrink to molehills as time flies past! How, instead of striving for our rights and privileges we should aim at hearing the Master say, "Well done, thou good and faithful servant!" But alas! we are human and forget to listen. But even from a business point of view a nurse should be so obliging that she earns what I heard said about a nurse the other day, "a real lady." "We always try to get nurse 'M,' we don't mind what we pay, she is never any trouble; only the greatest comfort."

### Wants and Workers.

Nurse Magan, 66 St. George's Square, S.W., would be very grateful for a second-hand self-propelling chair for a very poor man who is paralysed in the legs, to enable him to go out in the air.

### Where to Go.

Midwives' Institute and Trained Nurses' Club, 12 Buckingham Street, Strand.—Friday, March 29th, at 7.45. Post graduate lecture to midwives. Subject: "Hæmorrhage." Admission 1s. to non-members.

### Echoes from the Outside World.

AN OPEN LETTER TO A HOSPITAL NURSE.

At the Mansion House meeting on Tuesday it was decided that the national memorial to Queen Victoria should be erected on the site in front of Buckingham Palace, a decision which it was announced had previously been approved by the King. The Prime Minister, Sir H. Campbell-Bannerman, and others sent letters of apology for their absence, as their respective doctors had forbidden them to go out, owing to influenza and other causes. Mr. Balfour, in his speech, laid stress upon the fact that, "whatever else the memorial was to be, it must have nothing about it of a sectarian or political character, nothing which should not include among the subscribers men of every race, religion, creed, and political view among her late Majesty's subjects," and he appealed to the citizens of London to follow the example which had been so generously set by the citizens of Calcutta, who had already raised £250,000 for a similar object. After a speech by Sir William Harcourt, who described himself as one of the oldest servants of Queen Victoria, whose happy fortune it was to "look back through the golden epoch of Queen Victoria, from the bright dawn of the maidenhood of the late Queen to that noble end attended by the sorrow and love of her subjects." Several subscriptions were read out, including 1,000 guineas from the King, 5,000 guineas from the Corporation of London, £2,000 from Messrs. Rothschild, and £1,000 each from Sir Blundell Maple and Sir Thomas Lipton.

Though the fact that General Botha had refused the peace terms which Lord Kitchener offered him caused universal regret, it occasioned no surprise until the end of last week, when the particulars of the terms were made public. Then, except among people who can see no virtue in anything English, the feeling was one of wonderment that the Boers should have manifested such shortsighted policy, so exceptionally and unexpectedly favourable were the conditions put forward. The only reason which can be assigned for the refusal is that the very generosity of the terms may possibly have been regarded by General Botha and his colleagues as a sign of weakness, and as showing how exceedingly anxious we are to end the war. So we are, but not only in our own interests. It is partly that the country may have a speedy opportunity of recovering itself, that the farms which are at present unburnt may be saved, that needless suffering and privation of the Boer women and children may be prevented. But, strange as it sounds, these points which one would have fancied would have weighed considerably with men who call themselves brave patriots appear to be of no account, and all the miserable warfare is to go on until even the remnant of the hostile force shall have been scattered or destroyed. Unhappily, but, of course, it will be impossible to offer again such good terms as have just been spurned, at least as to the personal treatment of those who prolong a hopeless and ruinous war, or in regard to the question of a special war tax. But it is believed that the outline of the scheme which Lord Kitchener unfolded to the Boers will ultimately be carried into effect. Independence for the rebels cannot be acceded in any form, because they have proved themselves not to be trusted, but there will be a Governor and an Executive Council consisting of a certain number of official members and a certain number of unofficial members. Later on a "representative element" will be introduced. But just now the realisation of these schemes of peace seems far ahead.

This is the Census week, and ever since Monday morning 40,000 enumerators have been busy at work leaving the formidable blue papers at every house in the United Kingdom and the Channel Islands. In some of the poorer districts where a good many people live in one room the enumerators have decided not to begin their distribution until Saturday, lest the papers should be mislaid or destroyed before the fateful thirty-first arrives. According to the directions given to each householder the details are to be filled in on the morning of Monday, April 1st, but there are objections to these orders being carried out to the letter other than the rather foolish one of the date being "All Fools' Day," when it is considered by some legitimate to do or to write untruths for the "fun of the thing." The fact of £5 being the penalty for "wilfully giving false information" will, I fancy, deter most householders from playing jokes, but in cases where a man goes off to work at 5 A.M. it is manifestly impossible to do a quarter of an hour's thoughtful writing before starting forth, so that it may be safely predicted that in nine out of every ten houses in the kingdom the census papers will be finished off, if not entirely filled up, on Sunday.

Having regard to the difficulties of ignorant or uneducated householders to whose lot it may fall to tackle what to them will be as bad as a Chinese puzzle, the teachers at many, if not all, of the Board Schools are having a "census class" throughout the week. They take a census paper and fill it up in a correct manner so that all the children may read and note, and are thus able to assist substantially their less favoured parents. In Wales special schedules printed in the Welsh language have been provided, and in Whitechapel and elsewhere where Jews abound Yiddish-speaking distributors have been employed so that they may be of as much service as possible to enquirers who are anxious not to spoil their papers. Many letters have been sent to the Census office to request that, if possible, the writers may be allowed to return the schedules direct to the office instead of to the distributors, because they so strongly object to local tradespeople or workmen knowing all their private affairs. But it is hardly necessary to say that these good folks have made their protest in vain. More care has been taken this time to discover what proportion of the population is defective in mind, sight, hearing, or speech. The estimated cost of numbering the people will be about £187,800 it is believed, against only £91,900 ten years ago. The price paid to the collectors for every hundred names over the first 400 has been raised from 2s. 6d. to 3s. 6d. a hundred, which may account a little for the extra cost, but the increase in the population is the principal feature, notwithstanding that there are 500 fewer births daily in the United Kingdom than twenty years ago. For the first time women clerks are employed in the tabulation.

By the death of Miss Charlotte Yonge, which took place on Sunday, the world is distinctly the poorer. Though not a great writer, she had the literary faculty strongly developed, and one of her most conspicuous gifts was the power of imparting, to young people especially, the historical knowledge with which it may fairly be said she was saturated. If she had written only "Cameos from English History" she would have established a substantial claim to originality of treatment; but her versatility was considerable, and among the many admirable novels which she published in the course of her tranquil life "The Heir of Redclyffe," "The Daisy Chain," and "The Little Duke" have enjoyed a large and well-earned measure of popularity. A devoted Churchwoman, whose teaching was always good but never "goody-goody," she lost no opportunity of showing her interest in the missionary cause, to which she not only liberally contributed in money, but also in literature. Her memoir of Patteson, the martyred Bishop of Melanesia, is an enduring evidence of her sympathy with the labours of those who lay down their lives for the sake of their religion. She was herself an active worker in the pretty parish of Otterbourne, where she resided, and those who knew her personally were warmly attached to her. She has passed away at a good old age, leaving behind her the fragrant memory of a woman who not merely succeeded in gaining the ear of the public, but did something to make the world better than she found it.

### A Book and its Story.

### THE ODD GIRL IN A FAMILY.*

THAT the story of Anne Mainwaring, the odd girl in a family, where other sisters were acknowledged beauties and society successes, is intensely interesting no one who reads it can deny. There is a directness that appeals to one, and a reserve and refinement in depicting certain scenes which in the hands of a less artistic writer might have been otherwise. Ethically, to many readers, the unsatisfactory ending of the book will come as a disappointment. For the interest grows with the story, and the vacillation of Anne's strong character under the pressure of an ordeal, which to one of her temperament must have been no ordinary trial, is difficult of comprehension. Had she not decided to tread the thorny path of honour without love in preference to the one in which love was united with dishonour? This being so it would have been eminently more satisfactory if the authoress had not left the reader as undecided as to Anne's future conduct as she herself apparently was. Perhaps it is intended that each shall decide the complex question in his own way as experience or intuition serves. But personally we lean to a comfortable conclusion definitely defined—if not necessarily agreeable to it ourselves.

"I can't think how she comes to be my daughter." In this opening sentence of the first chapter Anne is brought on the scene. Her mother raises the dubious query from the depths of her couch, conveniently arranged that the shaded light of a lamp may fall on the features, delicate if somewhat insipid, which helped her play the rôle of an invalid successfully.

As she lay there in her dainty lace tea-gown she gave the impression of something very fresh and youthful. It was difficult to believe that the two tall girls standing beside her could really be her daughters. "We three seem just like sisters," she was fond of saying to every new acquaintance. Mrs. Mainwaring had other weaknesses. She lacked perception. Being without sensitiveness herself, she failed to realise that others were not without feeling. On this particular afternoon she was enlarging on the points of difference between her daughters. These she discussed regardless that Anne was present. She was accustomed to ignore the fact that her careless words might wound . . . for no careless word or jest had ever ruffled the surface of her own self-approval; she merely said, without reflection, what occurred at the moment, what was most amusing or effective. "It's too extraordinary," she went on in her clear rather high pitched voice; "all the others are fair—fair as I am . . . they are all like that, all but Anne."

"Yes!" exclaimed a lady visitor, "you have lovely children."

"The whole duty of woman is to be pretty," said Mr. Mainwaring. "I quite agree with you," struck in another visitor, in an emphatic tone. "I even go further, and say that for the sake of future generations, the ugly ones ought to be prohibited from marrying. Think what a mercy it would be if those, who are badly handicapped to start with, were disqualified from the race altogether. The course is so terribly overcrowded, even with pretty women, as it is." Anne, from her ambush by the window, hears, and takes in this conversation unobserved. She realises, little more than a child as she is, that in her world there is no place for a girl not endowed with good looks; but the next remark, if not exhilarating, is suggestive.

"If I had an ugly girl, I should make her take to philanthropy, or cooking, or any other occupation she might have a turn for, and I should bring her up to believe that was her lot in life."

"We don't quite know what we are to do with Anne," Mr. Mainwaring went on with a heavy playfulness; "she certainly won't be a cook, and I should be sorry for the hospital where she was a nurse."

To poor Anne, her own lack of personal charm and beauty was a real sorrow. To her mother, whose one object was to marry her daughters well, it was a grievance. To Anne, when later she goes out into society, unwillingly dragged to balls where her sisters' partners take her only on sufferance, the position is almost intolerable. Possessing the sensitive perceptive temperament, with strong artistic instincts, she realises bitterly her own plainness as a discord. She longed for beauty, for beauty's sake; and she longed for appreciation and understanding, two things which by reason of her reserve and pride she was not likely to command. Compensation is found for their absence by her talent for art. Into this she throws herself after every obstacle has been raised in her way to prevent its exercise. In art, in work for art's sake, she hoped to forget chagrin and ennui. Love she fancied unnecessary to the woman with a career. But withal, at the back of everything, this was the one thing indispensable to a temperament like hers—the one thing which could really give softness and sweetness in place of hardness and asperity. Naturally many men are about her. Her father's and mother's guests included the eligible *partis* of the season. In the, to Anne, far-away days when she was fourteen, she had had a boy champion whose chivalrous instinct was stirred by her forlorn position. He had cheered and consoled her on one memorable occasion when, desperate at the taunts of her mother, she had broken away in a fury of rage and misery, and was found by him in a perilous position in the picture gallery, standing on a chair with a light in her hand, peering into the face of a grim ancestor, the one black man in a race of fair Mainwarings, whom she was said to resemble. This scene, in the uncertain light of a winter twilight, with a storm of snow and sleet raging outside, is quite one of the best things in the book in its way.

Among other guests when Anne is eighteen is George Lumley, a rising barrister, a good-natured, not unkindly man, with enough knowledge of the first principles of art to interest himself in Anne's painting. She confides in him her hopes and fears, and he helps her by advice and criticism. Lovers do not come to Anne, but Lumley's interest in Anne deepens into a desire to have her as his wife. She, at the earnest wish of her mother, accepts him, and their marriage is as successful as matches of the kind may be until the testing moment comes. Anne had become indifferent to her husband, whom she never really loved, their child being their only bond of union at the moment when she hears that Lumley had been her sister's lover, and had been rejected by her. This arouses a dormant feeling of injustice at not being told before her marriage of this. Then comes apathy, and then, alas! for her husband and child, comes the other man, her girlhood's champion. With his appearance the dramatic situation increases, not lessened by Anne's friendship with the very loveable but not blameless Lady Katherine Ashton. We hope that this faint outline of Anne Mainwaring's story will be of interest to those readers who may or may not have an opportunity of reading it for themselves: but of the new novels it is certainly one of the best.

* "ANNE MAINWARING." By LADY RIDLEY. (London: Longman and Co. 6s.)

### Everybody's Opinion.

[Correspondence on all subjects is invited, but we cannot in any way be responsible for the opinions expressed by our correspondents. No communication can be entertained if the name and address of the correspondent is not given, as a guarantee of good faith but not necessarily for publication, or unless one side of the paper only is written on.]

### THE QUEEN AND THE ROYAL NATIONAL PENSION FUND FOR NURSES.

"Five Members of the Royal National Pension Fund for Nurses" write from Newbridge-on-Wye, Radnorshire: We, who are members of the Royal National Pension Fund for Nurses, and who were amongst those who were entertained at Marlborough House and received our certificates from the hands of our beautiful and gracious and beloved "Princess of Wales" last July twelvemonth, are most anxious to know whether we still retain, with the Princess's armlet, the name by which we were then allowed to be known, viz., "Princess of Wales's Nurses," or, as we bear their initials on our badges, and their Majesties have consented to continue to act as our patron and president respectively, shall we be "Queen Alexandra's Nurses?" No words can express the deep feeling of loving respect and admiration which we—country nurses though we are—feel towards our—until lately—most sweet and gracious Princess, and now our beloved Queen. The day in which we saw her face to face, and received not only our certificates and badges but what we valued even more, her smile and welcome, will never be forgotten, though we may never again have the opportunity of seeing her. We would like to feel that we belonged to her personally still. Your intimation last week was the first notice we had seen that our Queen would still be our president. We had been waiting for some information on the subject.

### THE NURSES' CO-OPERATION.

"Miss Honnor Morten" writes from Ivy Hall, Richmond: I was very glad to see your word of warning about this co-operation. I was at the annual meeting, and it seemed to me that Mary Belcher with her quaint originality and charming courage had struggled in vain, and that nurses have merely exchanged the tyranny of the one head of the old institution for the tyranny of the two or three who now run a committee. I moved at the meeting that the co-operation should send a representative to the International Congress of Nurses at Buffalo, but there was not an atom of public or professional spirit amongst the women present, and I understand that the whole thing has been negatived without ever being referred to the nurses. The nurses are forced to join the Howard de Walden Club, and are herded there as at other institutions, and there seems to be no gain of freedom or independence, but all the old listlessness and powerlessness.

### TWO CONCEPTIONS OF THE NURSING VOCATION.

"B.P." writes: The experienced matron of some (most unfortunate?) institution made a most scathing summary of a nun's inability to make a good nurse in the Nursing Mirror of March 2nd. She wrote: "A nun is of choice an unsexed and unsympathetic nonentity, and therefore could never make a sensible, sensitive, or sympathetic nurse." My experience makes me think differently. I had the good fortune some few years ago to be nursed in a convent in Italy, and to my dying day I shall never forget the sympathy, the loving care, and the forgetfulness of self which the good nuns there displayed to a stranger and a Protestant. My opinion is that a nun, a nurse, or any other individual shows lack of breeding, and is less well treated, if she is perpetually on the defensive, and so afraid lest she should be "treated in a light or disrespectful manner." One usually sinks or rises to one's own level, and will be treated accordingly. The very dangerous and unsafe man who, probably not through ignorance of manners, but of nurses and nurses' habits, suggested that the nurse should dine in the housekeeper's room would most likely have apologised for his insult and want of gentlemanliness if she had given him the opportunity of becoming better acquainted with her.

### SISTERS AND PROBATIONERS.

"M. M." writes: "An Old Hand's" reply to Miss Twining's letter was intensely interesting, but rather too short. My first days of probation come vividly before me; I even fancy I can see the graceful form and hear the loud voice of my charge nurse. Not one patient liked her: she seemed to act upon one and all like a wet blanket, "as it were." One probationer went out for a walk and never returned on her account. I well remember one day I was, as I thought, all alone in the nurses' sitting-room: the piano stood open, and feeling quite safe I began playing a few bars. "Close the piano at once; we don't allow 'pros' to touch it until they have been here twelve months." I turned round, and there stood the charge nurse in a very commanding attitude. As a paying probationer I never thought I should be treated in such a manner. One evening we were startled in the midst of tea. "I met Dr. —— in the City to-day, and what do you think he said? 'Really, nurse, you get more graceful every day.'" She suddenly left the table and walked to the opposite mirror, twisted, turned about, coming away very satisfied with herself. She once called out to me from one end of the ward to the other, "You are not right." I believe, poor thing! she came on this earth fully trained and wearing indoor uniform. Nevertheless, I was taught a golden lesson: should I ever become a charge nurse to remember my own days.

"E. D." and "H. S." write: We quite agree with "C. F. Y." as to the harassing of the poor probationers. We think they have far too much menial work. We know of some probationers who have been in "training" for about eight months who have no more knowledge of taking temperatures and marking charts than a baby has. But what can one expect when, agreeing for two years, or three, as the case may be, before knowing anything about the hospital, &c., they are put to train under a woman, called "sister," who has herself had no training except such as a very small fever hospital affords? Why people advertise for "gentlewomen only" and treat them worse even than domestic servants we cannot imagine. It is not very pleasant for any nurse who has had thorough good training in all branches of infectious diseases, and through no fault of her own not able to go for general training, after being in a hospital for any length of time, to make a change and discover to her dismay that she has been "taken in." For instance, she may be and has been put under an untrained "sister" who knows practically nothing about the profession above taking temperatures and pulses and respirations and other like small items; as for tracheotomy she has never seen a case, and does not know what a mastoid abscess is. All goes on well for the poor assistant nurse, if she is conscientious and reliable, until she finds the home, the rules, which differ from day to day, and the off-duty time, so uncomfortable that she resigns her appointment. Well! as soon as she sends in her resignation, she is put on "night" duty, and the "so-called" sister informs her she must do the "darning" of the ward, which is not a night nurse's duty, and is very bad for the eyes. Surely if a nurse is unable to read at night she is unable to darn. Then in the morning, because nurse happens to make a "pocket" a little awry, the sweet sister comes and strips all the beds, and the poor tired night nurse has to make them over again. One does not mind these things so much from a trained sister, but coming from a woman without training they go against the grain very much. The nurse who gave the utmost satisfaction before she resigned is now told "she cannot dust a ward or make beds properly." Did anyone ever hear of such a thing as a matron's word being "law" to the chairman of the committee? Did anyone ever hear of giving patients suffering from typhoid fever in the second and third weeks uncooked apples to eat, and oranges to peel and eat without even taking the pips out? We have known of such cases, and of course an assistant nurse must not dare to question what a sister does, whether trained or not, because she is "sister." Matrons and sisters ought not to treat a nurse spitefully just because she has sent in her resignation. Still, we are not judging all, for we were both trained under good sisters and matrons. We think that if a matron wants to keep up the name of her hospital, she should treat her nurses as she would like to be treated herself if she were a nurse, more especially a nurse who resigns, so that she take a good impression away with her.

### THE TRAINING OF INSTITUTE NURSES.

"A Trained Nurse" writes: It is high time the public were roused to the fact of how they are being duped by so-called nurses' homes. Picture, for instance, one home where there may possibly be about thirty nurses. These nurses altogether pay in a snug little income of over £300 a year to the matron. Trained nurses, they are called; but are they trained? Some few undoubtedly are and certificated; others, again, have maternity training only, never having been inside a general hospital or infirmary; others have passed some months possibly in a fever hospital; and yet others have passed a few months or weeks in a general hospital or infirmary—perhaps not even that. But so long as they don the nursing garb and pay in their fee monthly to the matron, and pay their board when they are in the Home waiting their turn for cases they pass as trained nurses. All these nurses, whether certificated or otherwise, take out printed rules with the fees charged for different diseases, and all demand the same fee and get it as a rule. Is this fair either to certificated nurses or to the public? What use is it for nurses to be properly trained in hospital or infirmary if women without any training are allowed to take the same fees? The maternity nurses, when disengaged, fill in their time with any case that offers, typhoid, or scarlet, or other diseases; then, after a week or so of quarantine, for which two guineas or thirty shillings is charged to the patient, they are allowed to again resume maternity work. Nurses of a few months' training will dare to go out to the most critical cases without the slightest knowledge of what they are called upon to do for such cases. Is it any wonder that patients slip through their fingers so easily? And in consequence of such inefficient workers the reputation of the Home suffers, the nurses lose work, but the income of the matron of the Home goes steadily on.

### NURSING AT CAPETOWN.

"Delta" writes from Capetown: Since I sent in my last paper and its publication in the *Cape Register* with that of an army sister who evidently has only been out here a little while, I have been busy collecting as much subject matter as I possibly can for the guidance and information of nurses in the old land, who, as I said before, might be dazzled by the £60 per annum offered by some of the nursing institutions in this colony. I shall be glad if I can only be the means of preventing one or two really good women from coming here to face utter disappointment and despair. Very few trained women possess sufficient private means to do as "Army Sister" suggests, and wait until they become known to the doctors. If they did they would soon find it melt away. My experience is that the doctors prefer to employ new comers before they get to know the perplexing and false ways of the colony, and readily offer them cases when they first arrive. It is when they have been here a short time that the struggle commences, and "the cares of bread" press heavily upon the workers. Possibly they have had trying, long cases, and are feeling the need of a rest themselves, and they are wanted by doctors because they cannot get others. To refuse would be to offend, to undertake cases means failure of health, perhaps collapse altogether; and when nurses fail in health here there is nothing but a going steadily to the wall, as I have seen far too many of such cases, and no one has cared for them, excepting to suggest that half a loaf is better than none, and advise them to take any fee they can get. In their perplexity and despair "they have consented, only to find that they could not live on the pay offered them, and finally they have disappeared. I know a nurse now, who, after being attached to a nurses' home for five years, is quite crippled, and doing needlework for such a living as she can make out of it. On the other hand I know of several pretty, inexperienced young women who are playing at nursing in the military hospitals at good fees, and boasting that they have nothing to do. I think that the fees for such valuable services to the Imperial Government are £2 2s. weekly. These, of course, are the women of the colony, with influence behind, and to suggest that they should be sent to their homes and families, and skilled nurses appointed in their places, would be to raise a storm of indignation in high places. I hear also, on good authority, that all sorts and conditions of women were employed to nurse on the transports. When one thinks of the tender, skilled nursing that typhoid patients require, and the numbers there are in the hospitals out here, one wishes one could renew one's youth and go and help, but, alas, we are not required. We must all be young in these days, and if handsome all the better. However, the bubonic plague is here now, so perhaps some of the older women will be wanted to nurse it."

### Appointments.

Ampthill Workhouse Infirmary.—Miss Annie E. Smith has been appointed charge nurse. She was trained at Tottenham Hospital, and has since been charge nurse at Islington Infirmary; nurse at the Infectious Diseases Hospital, under the Tendring Rural District Council; and nurse under the St. Albans Diocesan Institution for Trained Nurses.

Bucklow Union, Knutsford.—Miss Minnie Davies has been appointed assistant nurse. She was trained by the Meath Workhouse Nursing Association at St. Peter's Home, Kilburn, and has been assistant nurse at Maidenhead Infirmary.

Convalescent Home, Skegness.—Miss Valetta Shout has been appointed matron. She was trained at the Alexandra Hospital, London, and Addenbrooke's Hospital, Cambridge. She has been private nurse at the Nurses' Institute, Canterbury, staff nurse at the Sanatorium, Hull, and charge nurse at the City Hospital, Grafton Street, Liverpool.

Cork Street Hospital, Dublin.—Miss Bridget Faby has been appointed staff nurse. She was trained at the Cork Street Hospital, and St. Vincent Hospital, Dublin, and has since been assistant nurse at the former institution.

Croydon General Hospital, West Croydon.—Miss Ella M. North has been appointed night sister. She was trained at the Hospital for Diseases of the Heart and Paralysis, Soho Square, the Great Northern Central Hospital, and Queen Charlotte's Lying-in Hospital. She possesses the A.C.H. and the L.O.S. certificates.

Hastings Union.—Miss Mary Roberts has been appointed assistant nurse. She was trained by the Meath Workhouse Nursing Association at St. Peter's Home, Kilburn.

Kettering and District General Hospital.—Miss E. Ward has been appointed matron. She was trained at the London Hospital, and has since been staff nurse in that institution, and charge nurse at Kettering and District General Hospital.

London Central Schools' Infirmary, Hanwell.—Miss L. White has been appointed superintendent nurse. She was trained at the Infirmary, St. George's-in-the-East, where she was awarded the silver medal. She has since been staff nurse at the same institution.

Oxygen Home, Fitzroy Square, London. — Miss K. A. Smith has been appointed matron.

Plymouth Union Infirmary.—Miss Adelaide Christina Moffat has been appointed sister of the male wards. She was trained at the Buckingham General Infirmary, Aylesbury. She holds the L.O.S. certificate. Miss Dorothy Green has been appointed superintendent of night nurses. She was trained at the London Homœopathic Hospital, Great Ormond Street, W.C. For the last sixteen months she has been connected with the West of England Nurses' Co-operation, Plymouth.

Royal Edinburgh Hospital for Sick Children.—Miss Kathleen Lucretia Burleigh has been appointed matron. She was trained at St. Bartholomew's Hospital, London, and has since been staff nurse and acting night superintendent in the same institution; assistant matron at the Poplar and Stepney Sick Asylum; and matron at the Fountain Fever Hospital, Tooting.

### For Reading to the Sick.

HOLY WEEK.

Thus everywhere we find our suffering God,
And where He trod
May set our steps: the Cross on Calvary
Uplifted high
Beams on the martyr host, a beacon light
In open fight.

To the still wrestlings of the lonely heart
He doth impart
The virtue of His midnight agony,
When none was nigh,
Save God and one good angel to assuage
The tempest's rage.

"O Father! not My will, but Thine be done!"
So spake the Son.
Be this our charm, mellowing earth's ruder noise
Of griefs and joys;
That we may cling for ever to Thy breast
In perfect rest!—*Keble.*

That cry from the cross was but the audible expression of His whole life and death; the concentrated utterance of all His sorrows, all His acquaintance with grief. "Father, forgive them!"—that was what it all meant. And, therefore, that He made "intercession for transgressors," is the conclusion of all this story of sorrow and grief.

And "Father, forgive them" is the utterance and meaning of His whole heavenly life in human form, even as of His earthly; for what He began below, He is continuing above. We have spoken of Him before as, in a sense, a Man of Sorrows, even now. And still does He appear as a Lamb slain, standing before God for us, "marred more than any man."

Oh, brethren! if our prolonged consideration of Christ's sorrows has at all led us to realise them, this must be a terrible thought for us, seen in this light, the imperfection of our spiritual life. Yet let us take comfort in the remembrance that those very sorrows of His are the great pledge that prayers and efforts after "more life and fuller" shall not be in vain; they form the ever-availing reason why "all things that pertain to life and godliness" should be granted to us, through Him Who came "that we might have life, and might have it more abundantly." And He still sorrows to that end.—*St. Hill Bourne.*

"Glory on glory compasseth Him round,
From henceforth unto all the deathless years;
The smile of God, wherewith He sitteth crowned,
More sweet, because the memory of tears
Is in His heart, and dieth not away;
And in exchange for every weary day
He spent on earth, some blessèd soul forgiven,
Some face once darkened with our sin and night,
Is lifted up to Him in cloudless light,
And addeth glory to those days of heaven."
*From the "King of Sorrows."*

### Presentations.

Oxygen Home, Fitzroy Square, London.—The committee of the Oxygen Home presented the matron, Miss McCotter, on the occasion of her departure for South Africa, with a beautiful morocco dressing-case, containing a complete set of very handsome solid silver fittings. On the inside of the lid a silver plate bore the following inscription: "Presented to Miss McCotter by the Committee of the Oxygen Hospital, in grateful appreciation of the valuable services to the Institution. March, 1901." Miss McCotter has resigned her post to join the Army Nursing Service Reserve, and sailed from Liverpool on Monday last.

### Notes and Queries.

**The Editor is always willing to answer in this column, without any fee, all reasonable questions, as soon as possible.**
**But the following rules must be carefully observed:—**
**1. Every communication must be accompanied by the name and address of the writer.**
**2. The question must always bear upon nursing, directly or indirectly.**
**If an answer is required by letter a fee of half-a-crown must be enclosed with the note containing the inquiry.**

*The Nurses' Union.*

(297) Would you kindly give me any information about the "Nurses' Union," and can any nurse join it?—*Nurse F.*

Apply to Miss Dashwood, Bryanston Square, W.

*Sanitary Inspector.*

(298) Would you kindly inform me the best place to qualify for a sanitary inspector, and would you consider it a fairly good occupation for a young lady to take up?—*B. E. D.*

Write to the Sanitary Institute, 72 Margaret Street, W. The salaries of women inspectors vary from £80 to £185 in London, and from £60 to £100 in the provinces.

*Phthisis.*

(299) To whom should I apply in order to get an "indulgence passage" in a hospital ship to South Africa? I am a private nurse, and being threatened with phthisis I want to join a friend at once in Cape Colony.—*M. M. L.*

For particulars as to hospital ships you should apply the Hon. Secretary, Central British Red Cross Committee, 18 Victoria Street, London, S.W. The United British Women's Emigration Association, Imperial Institute, S.W., would also be able to help you to travel cheaply and safely.

*So-called Registration.*

(300) 1. Is the certificate of the M.B.C.M., Glasgow, recognised by the Nursing Association?—*A.S.* 2. How can I become registered in a trained nurses' directory or register?—*Agatha.* 3. Is it wise to register as a trained nurse, and what steps are necessary in order to do so? Is there any advantage in belonging to more than one nursing association?—*Unregistered.*

The characteristic of the above queries is the vagueness with which they refer to "registration" and "nursing associations," whilst A.S. does not trouble to give even the name of her training school. The Royal British Nurses' Association, 10 Orchard Street, W., is the only association which compiles a register of its members who are properly trained nurses residing in every part of the world. Particulars may be had from the Secretary. "Burdett's Official Nursing Directory" is, as its name implies, a directory of all trained nurses who satisfy the editor as to their *bonâ fide* training. Any trained nurse may have her name entered by applying to The Editor, "Burdett's Official Nursing Directory," 28 & 29 Southampton Street, Strand. "Unregistered" would not be accepted by more than one nursing association at a time.

*Toilet.*

(301) Where can I be instructed in chiropody, and what would be the cost? *Sister Anne* (2), Can you recommend a reliable book of digestion and digestible foods; and tell me what is the best way to keep the hair fair; and to cure blushing? *May* (3), Where can I obtain instruction in electrolyses for the removal of superfluous hair? *E. A. H.* (4), What hospital in London gives treatment by the X-rays for the removal of superfluous hair? *Distressed* (5), Can anyone give me the address of a reliable hair specialist.—*Sister Eva.*

The above questions do not all relate to nursing topics. With regard to "May's" request for a book on digestion, Mrs. Ernest Hart's "Diet in Sickness and in Health," price 3s. 6d. from the Scientific Press, would probably suit her.

*Massage.*

(302) 1. I am anxious to obtain a certificate for massage; where can I get the necessary instruction? 2. Has any reader a second-hand copy of the "Art of Massage," by Mrs. Creighton Hale for disposal?—*C. W.* Also (1), *E. D. W.*, *E. P. M.* (in Liverpool). *N. C.* (in Manchester, fee and time required). *A Constant Reader* (on the Continent), and *H. P.*

Massage is taught in London at the hospital for Consumption, Brompton; the Hospital for Epilepsy and Paralysis, 32 Portland Terrace, Regent's Park, N.W.; the National Hospital for the Paralysed and Epileptic, Queen's Square, Bloomsbury, W.C.; the West End Hospital for Nervous Disorders, 73 Welbeck Street, W.; and by the Society of Trained Masseuses, 12 Buckingham Street, Strand, as well as by numerous private teachers. The secretary of the Society of Trained Masseuses will give reliable information to intending students on all details.

*Situation.*

(303) I was nurse in a children's hospital for about six months, but had to leave on account of my health. I am quite strong again, and would be glad if you could tell me of a hospital where I could be trained without a fee; or of a situation as useful companion to an invalid. My age is 19.—*Muriel.*

If you will wait until you are 23, you will find plenty of institutions ready to train you, provided you are suitable. You might enter a children's hospital, or try a situation as nurse to a delicate child until then. See advertisements.

*Lysol.*

(304) Can you tell me the strength that lysol should be used for douches after confinement?—*B.C.*

1 per cent. is a strength commonly used.

### Standard Books of Reference.

**"The Nursing Profession: How and Where to Train." 2s. net; post free 2s. 4d.**
**"The Nurses' Dictionary of Medical Terms." 2s.**
**"Burdett's Series of Nursing Text-Books." 1s. each.**
**"A Handbook for Nurses." (Illustrated.) 5s.**
**"Nursing: Its Theory and Practice." New Edition. 3s. 6d.**
**"Helps in Sickness and to Health." Fifteenth Thousand. 5s.**
**"The Physiological Feeding of Infants." 1s.**
**"The Physiological Nursery Chart." 1s.; post free, 1s. 3d.**
**"Hospital Expenditure: The Commissariat." 2s. 6d.**
**All these are published by The Scientific Press, Ltd., and may be obtained through any bookseller or direct from the publishers, 28 and 29 Southampton Street, London, W.C.**

### Travel Notes.

By Our Travelling Correspondent.

### LXIX.—ONCE MORE IN ROME.

My desire in these short papers is to take you to the less well-known places of interest in Rome, leaving such obviously important points as churches and museums to the intelligence of the traveller, fortified by the company of the invaluable Baedeker, and to visit in spirit the various spots, be they palaces, prisons, bridges, or streets, that are full of human interest and bathed in the glamour of an historic past. To-day then we will consider the palaces, with their often gloomy exteriors, which have hidden from the public eye the still more grim and gloomy incidents that have taken place within. It was my good fortune to wander in Rome with one who was a mine of antiquarian and historic knowledge: not a house, not a street, not a church, but he knew the most intimate facts connected with it, and the lifeless stone seemed to respond beneath his foot and yield up its closely-guarded secrets to his sympathetic spirit. To walk in Rome with him was to live in the absorbing past.

### The Palaces.

Let us begin with Palazzo Cenci-Bolognetti, which lies close to the old Ghetto and the beautiful Fountain of the Tortoises. Surely no story wrapped still in obscurity and overlaid with tradition and exaggeration can be more heart-rending and sickening than that of the hapless Beatrice Cenci, who probably in her short life of seventeen years never knew one single joyous hour, free from care, fear, and shuddering anticipation. The gloomy old palace seems to agree well with the frightful tragedies that stained its walls at the will of that monster Francesco Cenci. One can only hope and think that such abnormal wickedness could belong alone to a disordered brain.

### The Palace and Family of the Cenci.

It is very difficult in recording the lives of this ill-fated family to separate the true from the false, the length of time that has elapsed since the events, the absence of any public records of such startling events, and the enormous wealth and power of the family involved worked together to obscure the truth, and very contradictory accounts of the series of ghastly tragedies which resulted in the almost total extinction of the line of Cenci are met with in the different chronicles of the time. The story, roughly speaking and robbed of much that is not fit for publication, is this: Francesco Cenci, a monster who in these days would be restrained as a lunatic, after disgracing his house with every kind of enormity and being more than once imprisoned but bought off with enormous bribes, married as his second wife Lucrezia Petroni, who was childless. By his first wife he had several children, all of whom apparently he hated and treated with savage cruelty. His eldest daughter fled to the protection of the Pope, which enraged Francesco, and he persecuted Beatrice, his youngest child, as an accomplice. Somewhere about this time two of his sons were murdered, but by whom is not stated, and all is wrapped in mystery. Beatrice, then about sixteen years old, was imprisoned and beaten and treated by her father in a manner that will not bear repetition. It is sufficient to say that it was so monstrous as to suggest to her mind the thought of compassing his death. At that time (so irregular was the life of the clergy), Cardinal Guerra had been paying his addresses to her, and driven to desperation, the maddened girl asked his help and that of her stepmother, who loved Beatrice and was revolted by her husband.

To make a long story short, the two remaining brothers, with the connivance of Beatrice and Lucrezia, contrived his assassination: it was compassed by first drugging him and then driving a nail through his eye into the brain, after which the body was thrust through a window into the branches of a tree below with the idea that death should appear to have been by accident. The murder took place at Petrella, not far from Naples.

Several months passed, and possibly owing to Cardinal Guerra attempting to make away with the actual assassins, suspicion was excited. Eventually one of the murderers confessed, and after a short trial Lucrezia, Beatrice, and her two brothers were condemned and executed on the Ponte San Angelo. Among the many accounts given of this ghastly episode I have followed that of a French writer, Monsieur H. Beyle, in his *Chroniques et Nouvelles* as being on the whole the most fair and cool-headed. Immense efforts were made to save the victims but without avail, and all suffered on September 11th, 1599. The Cardinal escaped disguised as a peasant, and we hear no more of him. Beatrice was buried in front of the high altar in San Pietro in Montario.

### The Disputed Portrait of Beatrice.

We are all well acquainted with the reputed portrait of Beatrice in the Palazzo Barberini from the many inferior copies which flood the world. Prince Barberini refuses permission to set up an easel in front of this treasure; hence copies can only be made surreptitiously, I am told. For long this picture was supposed to be by Guido, but if it *is* in truth a portrait of Beatrice that is impossible, because Guido was only a very young boy at the time of her death; therefore if it is a Guido it is not Beatrice, and if it is Beatrice it is not a Guido. The romantic story of her turning her eyes on the painter in the crowd on her way to execution, and the other of his painting this picture in her cell at San Angelo on the night previous to her death, must be discredited. There seems no real proof of her ever having been imprisoned in San Angelo, though they show her comfortless bed there. Most accounts concur in giving the Palazzo Savella as her place of detention. I think Hawthorn's words descriptive of this very beautiful picture give the best idea of it I have ever heard expressed. It occurs in "Transformation." Hilda has been trying to paint the picture from repeatedly close observation, and thus describes her thoughts concerning it:—

"While I was painting her, I felt all the time as if she were trying to escape from my gaze. She knows that her sorrow is so strange and so immense that she ought to be solitary for ever, both for the world's sake and her own; and this is the reason we feel such a distance between Beatrice and ourselves, even when our eyes meet hers. It is infinitely heartbreaking to meet her glance and to feel that nothing can be done to help or comfort her; neither does she ask help or comfort, knowing the hopelessness of her case better than we do. She is a fallen angel—fallen, but yet sinless—and it is only this depth of sorrow, with its weight and darkness, that keeps her down upon earth and brings her within our view, even while it sets her beyond our reach. . . . It was terrible guilt, an inexpiable crime, and she feels it to be so; therefore it is that the forlorn creature so longs to elude our eyes and for ever vanish away into nothingness."—*Transformation.*

Such is the terrible record of that old time-worn palace near the Tiber over which hangs a grim and tragic mystery which will never cease to interest the world.

### TRAVEL NOTES AND QUERIES.

Rules in Regard to Correspondence for this Section.—All questioners must use a pseudonym for publication, but the communication must also bear the writer's own name and address as well, which will be regarded as confidential. All such communications to be addressed "Travel Editor, 'Nursing Mirror,' 28 Southampton Street, Strand." No charge will be made for inserting and answering questions in the inquiry column, and all will be answered in rotation as space permits. If an answer by letter is required, a stamped and addressed envelope must be enclosed, together with 2s 6d., which fee will be devoted to the objects of the "'Hospital' Convalescent Fund." Any inquiries reaching the office after Monday cannot be answered in "The Mirror" of the current week."

Abroad for Economy (Narrow Means).—Whether living is cheaper in France than in England is a question often asked, and the answer is this. Speaking generally, actual living is not cheaper, but house rent is lower if you are content to choose a small country place not frequented by the English and removed from the conveniences of large towns. The great difference lies in not having to keep up an appearance. Those who in England would have certainly two, and probably three, servants, in France will live just as comfortably with one if they do not mind helping a little themselves. The French do not value us according to our establishment, as is, alas! too much the case in England. Groceries are dear, and, I think, not so good as ours; meat, too, is not cheap; but poultry, eggs, and butter come cheaper, and vegetables and fruit are plentiful and inexpensive. But the saving is in our style of living.

Monte Carlo (Artist).—I should not advise your staying in Monte Carlo itself: it is very expensive and rather hot at this time of year. Why not get quarters at Mentone, or, if economy is a great object, at Ventimiglia? It is really very near to Monte Carlo, and for your especial purpose would, I think, be in every way preferable. I do not think that your terms would be accepted anywhere in Monte Carlo, whereas for that amount in Ventimiglia you would be comfortable. Your cycle would take you along the Cornice in half an hour to all kinds of fascinating corners.

